# The Effects of Group Arts Therapy Based on Emotion Management Training on the Emotional Expression, Alexithymia, Depression and Quality of Life in Patients with Schizophrenia

**DOI:** 10.1192/bjo.2025.10172

**Published:** 2025-06-20

**Authors:** Sang-Yeol Lee, Hye-Jin Lee

**Affiliations:** 1 Department of Psychiatry, Wonkwang University School of Medicine and Hospital, Iksan, Korea, Republic of; 2 Department of Public Health, Wonkwang Graduate School, Iksan, Korea, Republic of

## Abstract

**Aims:** The objective of this study was to investigate the effects of group arts therapy based on emotion management training on emotional expression, positive and negative emotion, alexithymia, depression and quality of life patients with schizophrenia.

**Methods:** 24 patients of 160 inpatients with schizophrenia from H Mental Health Care Facilities in G city were randomly assigned to either an experimental or control group. Each group were consist of 12 patients. Group arts therapy was conducted on the experimental group twice a week, 60 minutes per session, for a total of 16 sessions. The following scales were used for assessment: Berkeley Expressivity Questionnaire (BEQ), Positive Affect and Negative Affect Schedule (PANAS), Toronto Alexithymia Scale (TAS-20K), Depression Scale for Schizophrenia (K-CDSS), and Schizophrenia Quality of Life Scale (SQLS). Repeated measures ANOVA was conducted to confirm the differences for scores of each scales regarding groups, measuring timing, and also the interaction between groups and measuring timing.

**Results:** Group arts therapy increased emotional expression (total score, expressivity and impulse strength subscale socre of BEQ) in experimental group compared with control group (p<.001). Group arts therapy increased positive emotion (p<0.001) as well as decreased negative emotion (p<0.05) in experimental group compared with the control group. Group arts therapy increased score of difficulty identifying feelings of TAS-20 (p<0.01) in experimental group compared with the control group. Group arts therapy decreased depression (p<0.01) in experimental group compared with the control group. Group arts therapy increased quality of life (p<0.01) in experimental group compared with the control group.

**Conclusion:** The group arts therapy significantly improved the emotional expression and positive emotion, and decreased negative emotion, alexithymia and depression in patients with schizophrenia and also improved quality of life. These results suggest that group arts therapy based on emotion management training could be a useful intervention for emotional disturbance in patients with schizophrenia.

